# The risk factors and prediction model for postoperative pneumonia after craniotomy

**DOI:** 10.3389/fcimb.2024.1375298

**Published:** 2024-12-24

**Authors:** Bingbing Xiang, Mingliang Yi, Chunyan Li, Hong Yin, Shun Wang, Yiran Liu

**Affiliations:** ^1^ Department of Anesthesiology, Chengdu Fifth People’s Hospital (The Second Clinical Medical College, Geriatric Diseases Institute of Chengdu/Cancer Prevention and Treatment Institute of Chengdu, Affiliated Fifth People’s Hospital of Chengdu University of Traditional Chinese Medicine), Chengdu, China; ^2^ Department of Anesthesiology, West China Hospital, Sichuan University, Chengdu, China; ^3^ Clinical Medical College and The First Affiliated Hospital of Chengdu Medical College, Chengdu, China; ^4^ Nursing Department, Chengfei Hospital, Chengdu, China; ^5^ Department of Neurosurgery, Chengfei Hospital, Chengdu, China

**Keywords:** craniotomy, postoperative pneumonia, risk factors, prediction model, pathogens

## Abstract

**Background:**

Craniotomy is highly susceptible to postoperative pneumonia, which significantly impacts the outcomes of patients undergoing such procedures. Our study aims to examine the risk factors associated with postoperative pneumonia and establish a predictive model with a nomogram to assess this risk.

**Methods:**

We conducted a matched 1:1 case-control study involving 831 adult patients undergoing craniotomy at our hospital. Cases consisted of patients who developed postoperative pneumonia within 30 days after surgery, as defined by consensus criteria. Controls were randomly selected from a pool of eligible patients.

**Results:**

The overall incidence rate of postoperative pneumonia is 12.39% in a total of 831 surgeries, which associated with unfavorable outcomes. Gram-negative bacteria were found to be the most common causative agents and approximately 27.94% of cases attributed to multi-drug resistant strains. The logistic regression analysis revealed five independent risk factors, as follows: smoking history, surgical duration, postoperative albumin, unplanned re-operation, and deep vein catheterization. A risk prediction model was derived and a nomogram was constructed. The Hosmer-Lemeshow test yielded X^2^ = 3.871 (P=0.869), and the receiver operator characteristic curve analysis demonstrated an area under the curve of 0.898 (P<0.05), with a sensitivity of 79.6% and a specificity of 85.4%, indicating excellent model fit and predictive performance. In addition, the C-index of the nomogram model was 0.898(95%CI, 0.853~0.941). The calibration curves of the nomogram model showed p-values of 0.797 and the Brier scores were 0.127. The analysis of the clinical decision curve showed that the nomograph model had high clinical application value.

**Conclusions:**

Postoperative pneumonia patients after craniotomy exhibits distinct pathogen distribution and is strongly associated with unfavorable outcomes. The risk prediction model developed in this study demonstrates a good fitting degree and predictive performance. The constructed nomogram model is objective, specific, and easily applicable in clinical practice.

## Background

Craniotomy plays a pivotal role in the management of brain tumors, traumatic brain injury, cerebrovascular disease, and movement disorders (Renz et al., 2018). However, the extensive surgical trauma and prolonged bed rest associated with craniotomy make patients highly susceptible to postoperative pneumonia (POP), which significantly impacts their prognosis. Notably, secondary infections, including pneumonia, following traumatic brain injury are reported as leading causes of mortality (Wang et al., 2020). Therefore, this retrospective study analyzed the clinical data of patients undergoing craniotomy to explore the risk factors for postoperative pneumonia and develop a prediction model. Ultimately, this prediction model can guide neurosurgeons and anesthesiologists in early identification of high-risk factors and implementation of proactive interventions to prevent the occurrence of postoperative pneumonia.

## Methods

### Study design and participants

This study received approval from the hospital ethics committee (PJ202139) and encompassed a total of 831 patients who underwent craniotomy at the hospital between January 1, 2022, and June 31, 2022. The inclusion criteria consisted of adult patients who developed new-onset pneumonia within 30 days after craniotomy. Exclusion criteria encompassed patients with pre-existing pneumonia or those diagnosed with pneumonia during surgery, patients who abandoned the surgical procedure, patients who experienced intraoperative cardiac arrest or required rescue measures, and patients with severely incomplete medical records. The diagnostic criteria for pneumonia established by the US Centers for Disease Control were employed in this study (Abbott et al., 2018). These criteria, which rely on chest X-ray findings and clinical symptoms, are primarily designed for adult patients and offer comprehensive and objective assessments. Adhering strictly to this definition, we identified patients with postoperative pneumonia and assigned them to the POP group. Subsequently, controls were matched by surgical specialty and randomly selected at 1:1 from the remaining surgical patients without pneumonia and assigned to the non-POP group.

### Data collection

Patients’ medical records were meticulously collected and documented through the hospital’s digital system. The collected data encompassed demographic factors (such as age, gender, height, weight, alcohol consumption history, and smoking history), general condition assessments (such as the Glasgow Coma Scale [GCS] and American Society of Anesthesiologists [ASA] classification), pre-existing medical conditions (such as hypertension, diabetes, chronic obstructive pulmonary disease), auxiliary examination indicators (such as chest X-ray findings, serum albumin levels, hemoglobin levels), operative factors (such as surgical duration, emergency surgery, unplanned re-operation, surgical incision), anesthetic factors (such as anesthesia method, duration of mechanical ventilation, blood transfusion, duration of postoperative bed rest), and invasive procedures (such as deep vein catheterization, radial artery cannulation, and urethral catheterization). In addition, the study also documented the causative pathogens and the presence of multi-drug resistant bacteria (MDR), which was defined as non-susceptibility to at least one agent in three or more antimicrobial categories.

### Statistical analysis

Statistical analysis was performed using SPSS 25.0 and Rstudio software. For normally distributed and homogenous metric data, the mean ± standard deviation (x ± s) was used to represent the data, and the independent sample T-test was employed to compare between groups. For metric data that did not meet the normal distribution and homogeneity of variance, the median (interquartile range) was used, and the rank-sum test was utilized for group comparisons. Frequency was used to express all count data, and the X^2^ test was employed for group comparisons. All factors that showed statistical significance in the univariate analysis were included in the multivariate regression analysis using a binary logistic regression model. A risk prediction model and a nomogram model were established using Rstudio software. The fitting degree of the model was evaluated using the Hosmer-Lemeshow test, and the predictive performance was assessed using the receiver operating characteristic curve (ROC). The bootstrap method was used to repeat the sampling 1000 times for internal validation and use the C-index for evaluation. Furthermore, the prediction model was evaluated using calibration curves and DCA. A significance level of P<0.05 was considered statistically significant.

## Results

### Patient characteristics and causative pathogens

**Figure 1:**
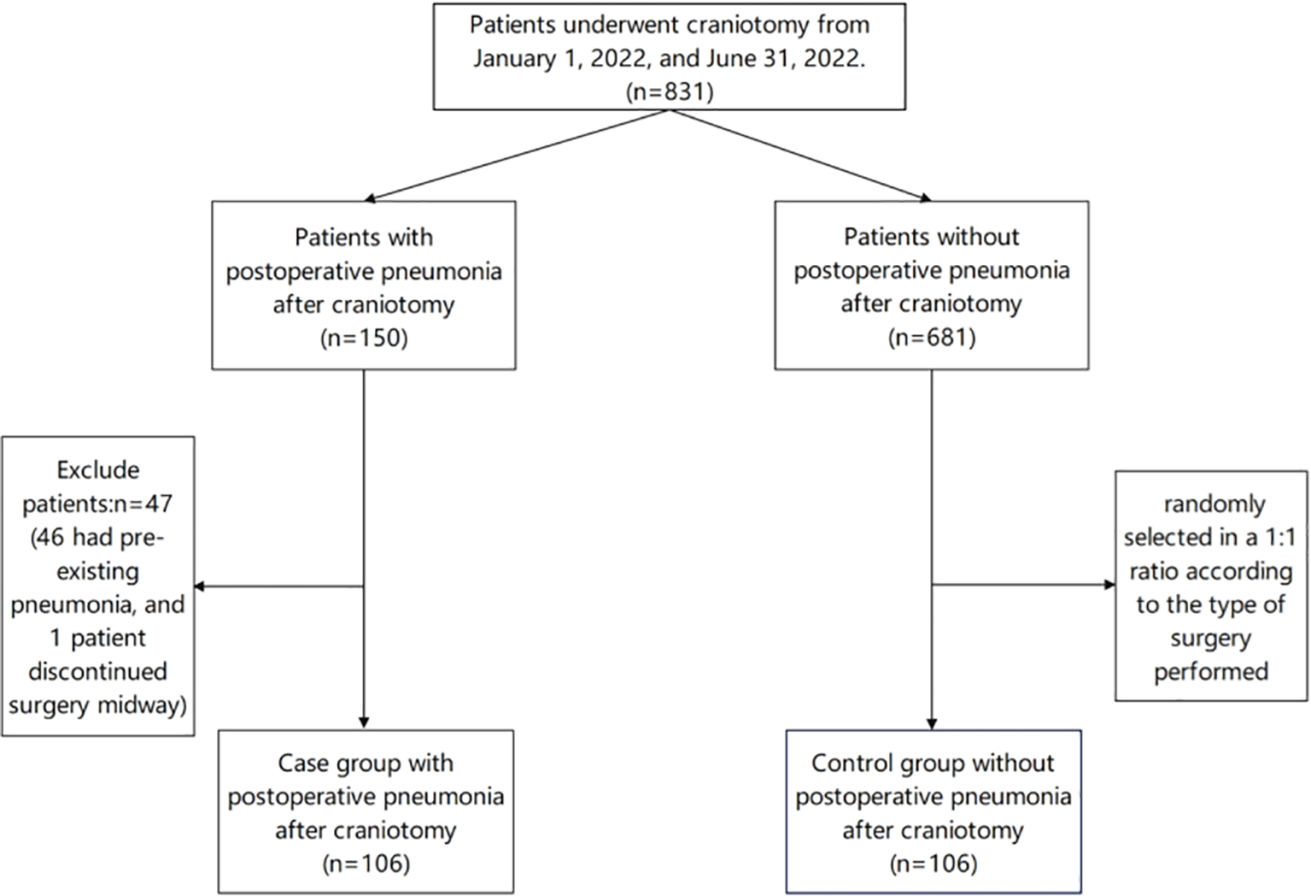
The flowchart of patient selection.

Within the scope of this study, a total of 831 patients who underwent craniotomy was examined. Among them, 150 patients were diagnosed with pneumonia, while 47 patients were excluded (46 had pre-existing pneumonia, and 1 patient discontinued surgery midway). Ultimately, 103 patients were diagnosed with postoperative pneumonia, resulting in an overall incidence rate of 12.39% (103/831). The flowchart of patient selection is shown in **Figure 1**. Co-culture analysis of sputum specimens from these patients revealed the isolation of 68 pathogenic strains. Among them, 51 strains were gram-negative bacteria, predominantly Klebsiella pneumoniae (26.47%), while 13 strains were gram-positive bacteria, mainly Staphylococcus epidermidis (13.24%). Additionally, 4 strains of fungi were identified, with Candida albicans (4.41%) being the most prevalent. Furthermore, 19 strains (27.94%) exhibited multidrug resistance, including 9 strains (13.24%) of carbapenem-resistant Enterobacteriaceae, 8 strains (11.76%) of carbapenem-resistant Acinetobacter baumannii, and 2 strains (2.94%) of carbapenem-resistant Pseudomonas aeruginosa. The distribution of pathogens is detailed in **Table 1**.

**Table 1:** Distribution of pathogens.

Pathogens	Strains (n=68)	Ratio (%)
Gram-negative bacteria	51	75.00
Klebsiella pneumoniae	18	26.47
Acinetobacter baumannii	10	14.71
Escherichia coli	5	7.35
Pseudomonas aeruginosa	4	5.88
Stenotrophomonas maltophilia	3	4.41
Burkholderia cepacia	3	4.41
Other Enterobacteriaceae	3	4.41
Burkholderia gladioli	2	2.94
Serratia marcescens	2	2.94
Citrobacter freundii	1	1.47
Gram-positive bacteria	13	19.12
Staphylococcus epidermidis	9	13.24
Staphylococcus aureus	4	5.88
Fungi	4	5.88
Candida albicans	3	4.41
Tropicalis	1	1.47

### Univariate analysis

**Table 2** presents the significant risk factors associated with postoperative pneumonia as identified through univariate analysis. Notably, 15 perioperative factors demonstrated a significant correlation with postoperative pneumonia (P<0.05). These factors include age, smoking, hypertension, diabetes, coma (GCS<8), ASA physical status, emergency surgery, postoperative hemoglobin, postoperative albumin, duration of ventilation, blood transfusion, surgical duration, unplanned re-operation, surgical incision, and deep vein catheterization. However, factors such as gender, body mass index, alcohol consumption, chronic obstructive pulmonary disease, preoperative hemoglobin levels, preoperative serum albumin levels, anesthesia method, radial artery cannulation, and urethral catheterization did not demonstrate a significant correlation with postoperative pneumonia (P>0.05).

**Table 2:** Univariate analysis of postoperative pneumonia in craniotomy patients.

Risk factors	POP group (n=103)	Non-POP group (n=103)	P
Age (years)	52.22 ± 14.57	45.10 ± 15.62	0.001
Age grading			0.007
<60 yr	73(70.87)	89(86.41)	
≥60 yr	30(29.13)	14(13.59)	
Sex			0.577
Male	49(47.58)	53(51.46)	
Female	54(52.42)	50(48.54)	
Body Mass Index			0.883
<24 kg/m2	68(66.02)	69(66.99)	
≥24 kg/m2	35(33.98)	34(33.01)	
Smoking History			<0.001
Yes	39(60.94)	72(69.90)	
No	64(62.13)	31(30.10)	
Alcohol History			0.063
Yes	35(33.98)	23(22.33)	
No	68(66.02)	80(77.67)	
Hypertension			<0.001
Yes	40(38.83)	17(16.50)	
No	63(61.17)	86(83.50)	
Diabetes			0.03
Yes	7(6.80)	17(16.50)	
No	96(93.20)	86(83.50)	
Chronic Obstructive Pulmonary Disease			0.071
Yes	96(93.20)	102(99.03)	
No	7(6.80)	1(00.97)	
Coma			0.002
Yes(GLS ≤ 8)	17(16.50)	3(2.91)	
No(GLS>8)	86(83.50)	100(97.09)	
Emergency Surgery			<0.001
Yes	31(30.10)	10(9.71)	
No	72(69.90)	93(90.29)	
Preoperative Hemoglobin			0.718
<110 g/L	5(4.85)	3(2.91)	
≥110 g/L	98(95.15)	100(97.09)	
Postoperative Hemoglobin			0.005
<110 g/L	44(42.72)	25(24.27)	
≥110 g/L	59(57.28)	78(75.73)	
Preoperative Albumin			0.447
<35 g/L	10(9.71)	7(6.80)	
≥35 g/L	93(90.29)	96(93.20)	
Postoperative Albumin			<0.001
<35 g/L	78(75.73)	49(47.57)	
≥35 g/L	25(24.27)	54(52.43)	
Anesthesia Method			0.718
General Anesthesia	98(95.15)	100(97.09)	
Local Anesthesia	5(4.85)	3(2.91)	
ASA Physical Status			0.025
<III	40(38.84)	56(54.37)	
≥III	63(61.16)	47(45.63)	
Duration of Ventilation			<0.001
<24 h	62(60.20)	98(95.15)	
≥24 h	41(39.80)	5(4.85)	
Blood Transfusion			0.007
Yes	17(16.50)	5(4.85)	
No	86(83.50)	98(95.15)	
Radial Artery Cannulation			0.118
Yes	98(95.15)	92(89.32)	
No	5(4.85)	11(10.68)	
Surgical Duration			<0.001
≤4 h	43(41.75)	83(80.58)	
>4 h	60(58.25)	20(19.42)	
Urethral Catheterization			0.490
Yes	91(88.35)	94(91.26)	
No	12(11.65)	9(8.74)	
Duration of Bed Rest			0.576
<3 days	49(47.57)	45(43.69)	
≥3 days	54(52.43)	58(56.31)	
Unplanned Re-operation			<0.001
Yes	48(46.60)	3(2.91)	
No	55(53.40)	100(97.09)	
Surgical Incision			0.023
Aseptic Incision	79(76.70)	64(62.13)	
Contaminated Incision	24(23.30)	39(60.94)	
Deep Vein Catheterization			0.004
Yes	82(79.61)	63(61.17)	
No	21(20.39)	40(38.83)	

### Multivariate regression analysis

For multivariate logistic regression analysis, postoperative pneumonia was defined as the dependent variable, while risk factors were considered as independent variables. The variables were assigned values with a significance level of α=0.05. The analysis revealed five independent risk factors for postoperative pneumonia after craniotomy (**Table 3**), as follows: smoking history (OR: 4.123 [1.619, 10.499], P=0.003), surgical duration (OR: 3.447[1.332, 8.919], P=0.011), postoperative albumin (OR: 3.525 [1.380, 9.002], P=0.008), unplanned re-operation (OR: 28.223[5.840, 136.406], P<0.001), and deep vein catheterization (OR: 5.766 [1.956, 16.996], P=0.001).

**Table 3:** Results of the multivariate regression analysis.

Risk factors	B	SE	Walds	P value	*OR*	95%CI
Smoking History	1.417	0.477	8.825	0.003	4.123	1.619, 10.499
Postoperative Albumin	1.260	0.478	6.936	0.008	3.525	1.380, 9.002
Surgical Duration	1.237	0.485	6.508	0.011	3.447	1.332, 8.919
Unplanned Re-operation	3.340	0.804	17.266	<0.001	28.223	5.840, 136.406
Deep Vein Catheterization	1.752	0.552	10.089	0.001	5.766	1.956, 16.996

### Risk prediction model

**Figure 2:**
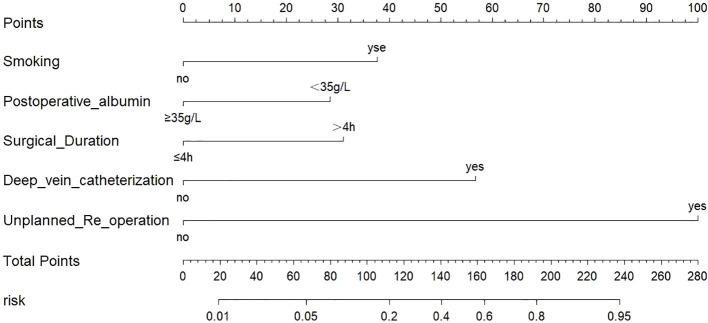
The nomogram model.

Based on the results of the multivariate logistic regression analysis, a risk prediction model using logistic regression was developed to predict the likelihood of postoperative pneumonia after craniotomy. The model’s predicted probability was calculated using the equation: logit (P) = 1/[1+e ^- 4.372 + 1.417× smoking + 1.260 × postoperative albumin + 1.237× surgical duration +1.752 × deep vein catheterization +3.340 × unplanned re-operation^]. The Hosmer-Lemeshow test confirmed that this model exhibited a good fit with the observed values (X^2^ = 3.871, P=0.869). A nomogram model, incorporating all independent risk factors, is presented in **Figure 2** for the prediction of postoperative pneumonia in patients undergoing craniotomy.

**Figure 3:**
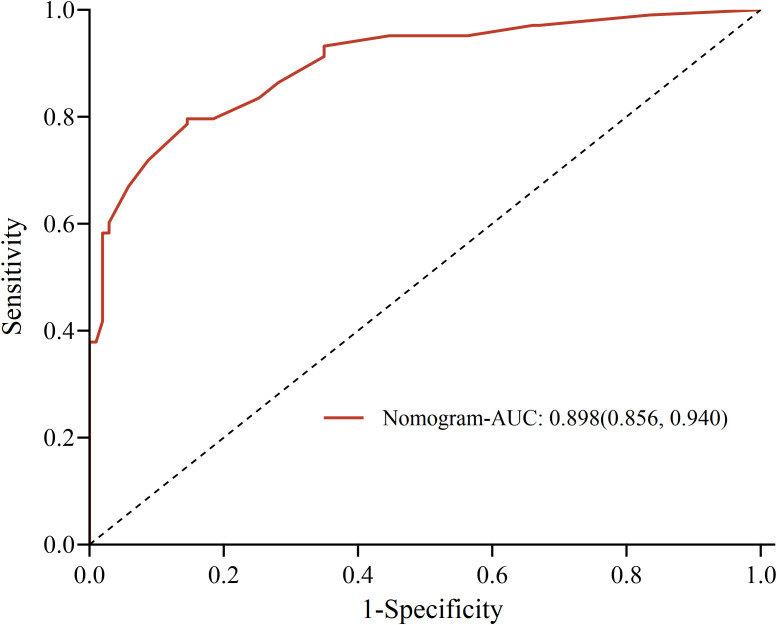
The receiver operator characteristic curve.

**Figure 4:**
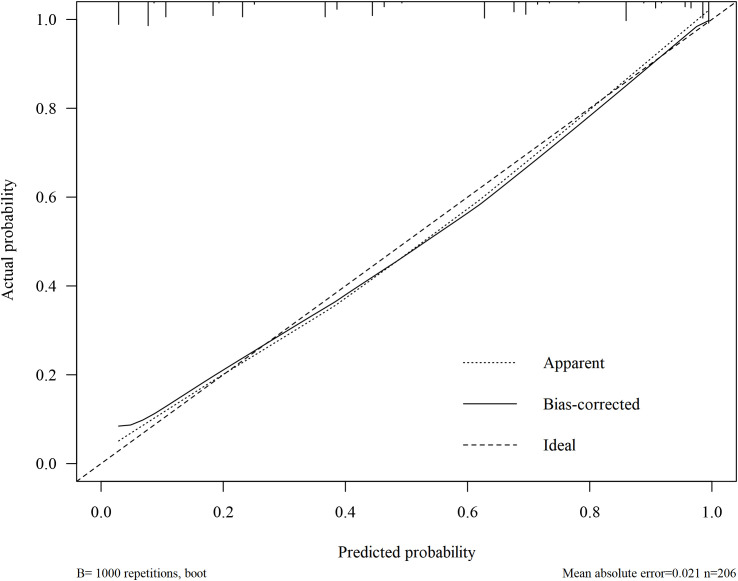
The calibration plots of the model.

The C-index of the model was 0.898(95%CI, 0.853~0.941). The greater the C-index was, the better the differentiation of the model was, indicating that the accuracy of nomogram prediction was great. The receiver operator characteristic (ROC) analysis revealed a remarkable area under the curve (AUC) of 0.898 (95% CI: 0.856-0.940), with a sensitivity of 79.6% and a specificity of 85.4%, indicating a commendable predictive performance, as shown in **Figure 3**. The calibration plots of the model showed that the nomogram had a good fit with the reference line, as shown in **Figure 4**, with p-values of 0.797, respectively (p > 0.05). The Brier scores were 0.127, respectively (close to 0), indicating that the prediction of the probability of POP after craniotomy by the nomogram model was consistent with the actual infection percentage of the observed population.

**Figure 5:**
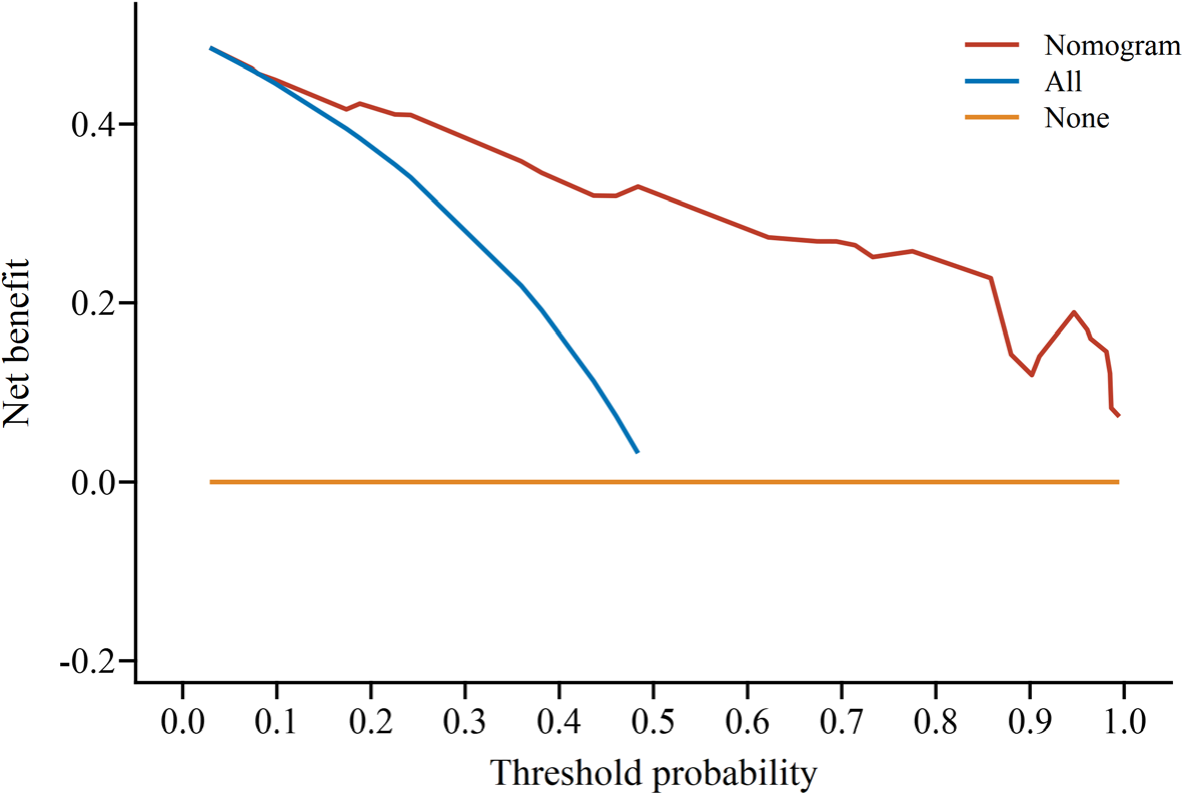
The threshold probability of the model.

The DCA determined the value of the clinical application of the nomogram model by calculating the net benefit under the POP risk threshold probability of each patient after craniotomy. The horizontal coordinate of the DCA was the threshold probability of high risk, and the vertical coordinate was the net benefit (NB). The high-risk threshold was set as (0, 1), and the net benefit rate and the range of effective pretest probability were assessed by subtracting the false-positive population that was incorrectly judged by the model. The threshold probability of the model set was between 0.01~1, all of which had a net benefit rate >0 and had clinical practical value, which suggested that the model had good clinical value in predicting POP after craniotomy, as shown in **Figure 5**.

### Outcomes

The retrospective analysis of outcomes of patients undergoing craniotomy with postoperative pneumonia is presented in **Table 4**. The findings demonstrated a significantly prolonged hospitalization duration in the POP group (28.09 ± 13.97) compared to the non-POP group (18.29 ± 7.13). Moreover, the proportion of neurosurgical patients requiring hospitalization for more than 14 or 30 days was significantly higher in the POP group than in the non-POP group (P<0.05). Additionally, the rate of ICU admission in the POP group was significantly higher than that in the non-POP group (P<0.05). Although there was no significant difference in the postoperative mortality rate between the two groups (P>0.05), it is worth noting that all three postoperative deaths occurred in the POP group.

**Table 4:** The outcomes of postoperative pneumonia following neurosurgery.

Outcomes	POP group (n=103)	Non-POP group (n=103)	P
Hospitalization Duration(days)	28.09 ± 13.97	18.29 ± 7.13	<0.001
≥14 days	51 (49.51%)	66 (64.07%)	<0.001
≥30 days	42 (40.77%)	5 (4.67%)	<0.001
ICU Admission	50 (28.54%)	8 (7.77%)	<0.001
Mortality	3 (2.91%)	0 (0%)	0.245

## Discussion

This study encompassed a total of 831 patients who undergoing craniotomy, with a notable incidence of postoperative pneumonia observed in 103 patients, accounting for approximately 12.39%. This incidence aligns with previously reported rates (Lo et al., 2017; Xiang et al., 2022). Notably, gram-negative bacteria remained the primary causative agent of postoperative pneumonia after craniotomy, constituting approximately 75%. However, the distribution of these pathogens exhibited unique characteristics, with Klebsiella pneumoniae (26.47%), Acinetobacter baumannii (14.71%), Staphylococcus epidermidis (13.24%), and Escherichia coli (7.35%) being the predominant strains. These pathogens are typically opportunistic bacteria and are commonly found in hospital environments as well as in the oral and pharyngeal cavities of patients, where they rarely cause infections. However, surgical trauma disrupts the integrity of the skin and tissues, creating an opportunity for these opportunistic pathogens to invade. Additionally, post-surgery, the patient’s immune function is suppressed, significantly diminishing their ability to combat pathogen infections. Furthermore, Staphylococcus epidermidis can be transmitted through hand contact, underscoring the importance of strict hand hygiene prior to invasive procedures such as tracheal intubation. Interestingly, this study revealed an incidence of approximately 27.94% for multidrug-resistant bacteria, with carbapenem-resistant Enterobacteriaceae (13.24%) and carbapenem-resistant Acinetobacter baumannii (11.76%) being the most prevalent. The emergence of bacterial drug resistance in patients after craniotomy poses a significant challenge, rendering clinical treatment more complex (Zhang et al., 2022). This may be attributed to the critical condition of patients after craniotomy, with some even presenting in a comatose state before surgery. Most patients after craniotomy exhibit compromised postoperative respiratory function and necessitate prolonged tracheal intubation. Once pneumonia ensues, it is almost invariably accompanied by polymicrobial infections, which may contribute significantly to the elevated prevalence of multidrug-resistant bacteria (Michalopoulos et al., 2005).

The multivariate analysis unveiled several independent risk factors for postoperative pneumonia after craniotomy, including smoking, postoperative albumin, surgical duration, unplanned re-operation, and deep vein catheterization. Leveraging the results of the multivariate logistic regression analysis, this study devised a risk prediction model. The model’s predicted probability was calculated as logit (P) = 1/[1+e ^- 4.372 + 1.417× smoking + 1.260 × postoperative albumin + 1.237× surgical duration +1.752 × deep vein catheterization +3.340 × unplanned re-operation^]. The Hosmer-Lemeshow test confirmed the model’s excellent fit and the ROC analysis validated its strong predictive performance. Furthermore, a visual nomogram was constructed to facilitate risk assessment for postoperative pneumonia patients after craniotomy, offering clinicians an intuitive tool for calculating the probability. The nomogram model, a graphical representation of clinical statistics, transforms complex multivariate analysis outcomes into a clinical risk assessment with segmented scales, providing objectivity, specificity, and obviating the need for extensive repetitive calculations (Wei et al., 2017).

Unplanned re-operation is a serious surgical adverse event, which significantly heightens the risk of infection and frequently leads to unfavorable outcomes (Longo et al., 2019; Zhao et al., 2022). It is estimated that approximately 53 to 69 million individuals worldwide suffer from traumatic brain injury annually (Dewan et al., 2018), and intracranial surgery substantially improves the survival rate of these patients (Pujari et al., 2018). In cases where patients undergoing intracranial surgery experience extensive cerebral infarction, delayed epidural hematoma, or secondary cerebral hemorrhage, an unplanned second craniotomy becomes imperative for life-saving purposes (Fu et al., 2023). Studies have demonstrated that smoking not only heightens the risk of pulmonary complications following surgery but also amplifies the likelihood of infection and in-hospital mortality (Fan Chiang et al., 2023). Smokers also experience prolonged hospital stays compared to non-smokers, with the detrimental effects of smoking persisting regardless of surgery duration or anesthesia technique. Our study findings have elucidated that emergency surgery independently contributes to an escalated risk of postoperative pneumonia, which is in line with the research conducted by McCoy CC et al (McCoy et al., 2015). Additionally, our study revealed a close correlation between prolonged surgical duration and postoperative pneumonia. Undoubtedly, the intricacy of craniotomy may contribute to extended operative durations, with soft tissue suturing and bone reconstruction further elongating the procedure. Moreover, Jing Yao et al (Yao and Liu, 2019). Observed that as the duration of the operation lengthens, the likelihood of contamination and infection increases, particularly for operations lasting more than 4 hours, which aligns with the outcomes of our study. Postoperative albumin emerges as an independent risk factor for the development of postoperative pneumonia (Viasus et al., 2013). A decrease in albumin can cause a decrease in plasma colloid osmotic pressure, leading to the retention of a large amount of fluid in the tissue gap, which is prone to pleural effusion and pulmonary edema, providing favorable conditions for the proliferation of pathogens. Studies have demonstrated a heightened incidence of pneumonia, prolonged ICU stays, poorer prognoses, and increased hospitalization costs among patients with hypoalbuminemia (Lim et al., 2012; Li et al., 2015). Given the critical condition and intricacy of patients undergoing craniotomy, a majority of them necessitate the placement of a deep venous catheter before surgery. Infection, particularly catheter-related bloodstream infections, represents the most prevalent complication associated with deep venous catheters (Teichgräber et al., 2011).

## Conclusions

Postoperative pneumonia after craniotomy patients exhibits a high incidence rate and is influenced by numerous perioperative risk factors. Moreover, it’s strongly associated with unfavorable outcomes. The pathogen distribution of postoperative pneumonia demonstrates distinct characteristics, including a prevalence of multi-drug resistant bacteria. The risk prediction model developed in this study demonstrates a good fitting degree and predictive performance, with the constructed nomogram model offering an objective, specific, and user-friendly approach. These findings support the proactive utilization of this model in clinical settings to identify high-risk patients early and implement timely intervention measures, thereby effectively reducing the occurrence of postoperative pneumonia and enhancing the overall outcome of patients after craniotomy.

## Data Availability

Data are available from corresponding author upon reasonable request. Requests to access these datasets should be directed to 2401900316@qq.com.
